# Active Learning for Efficient Soil Monitoring in Large Terrain with Heterogeneous Sensor Network

**DOI:** 10.3390/s23052365

**Published:** 2023-02-21

**Authors:** Hui Chen, Ju Wang

**Affiliations:** 1 Department of Computer & Information Science, CUNY Brooklyn College, Brooklyn, NY 11210, USA; 2 Department of Computer Science, CUNY Graduate Center, New York, NY 10016, USA; 3 Department of Computer Science, Virginia State University, Petersburg, VA 23806, USA

**Keywords:** soil, sensor networks, mobile sensing, active learning, machine learning, path planning

## Abstract

Soils are a complex ecosystem that provides critical services, such as growing food, supplying antibiotics, filtering wastes, and maintaining biodiversity; hence monitoring soil health and domestication is required for sustainable human development. Low-cost and high-resolution soil monitoring systems are challenging to design and build. Compounded by the sheer size of the monitoring area of interest and the variety of biological, chemical, and physical parameters to monitor, naive approaches to adding or scheduling more sensors will suffer from cost and scalability problems. We investigate a multi-robot sensing system integrated with an active learning-based predictive modeling technique. Taking advantage of advances in machine learning, the predictive model allows us to interpolate and predict soil attributes of interest from the data collected by sensors and soil surveys. The system provides high-resolution prediction when the modeling output is calibrated with static land-based sensors. The active learning modeling technique allows our system to be adaptive in data collection strategy for time-varying data fields, utilizing aerial and land robots for new sensor data. We evaluated our approach using numerical experiments with a soil dataset focusing on heavy metal concentration in a flooded area. The experimental results demonstrate that our algorithms can reduce sensor deployment costs via optimized sensing locations and paths while providing high-fidelity data prediction and interpolation. More importantly, the results verify the adapting behavior of the system to the spatial and temporal variations of soil conditions.

## 1. Introduction

This paper addresses the challenge of continuously monitoring soil conditions, such as chemical, physical, and biological properties. Soil is a thin layer of biogeochemically altered rock or sediment on the planet’s surface [1]. It supports ecosystem services critical for life, ranging from provisioning services, regulating services, to culture services [1,2,3]. Human activities, particularly agriculture, dramatically altered soil qualities, resulting in domesticated soils. Domesticated soils, with the removal of natural fauna, reduced biodiversity, and physical disruptions, are often unable to maintain the qualities of their original conditions that are essential to allow them to provide critical ecosystem services [1]. As a result, food security, climate change, and human health are among the global problems that demand us to monitor soil health.

Complementing traditional soil survey methods, sensor networks become a promising approach to observing soil in the field [4,5,6,7]. There are a wealth of published works focusing on sensor deployment to provide guaranteed coverage [8,9]. These works address the problem from the perspective of planning and are to determine the locations of the sensors in the field. Some researchers assume that sensors can sense an extended disk-shaped area to simplify the deployment problem [4,8,9,10]. There are several drawbacks of the coverage-oriented sensor location planning. First, many sensors are required to achieve full coverage in a practical use case, and consequently a high cost to acquire, maintain and operate these sensors. Second, the planned sensor networks are often “rigid” regarding the sensing service provided. End users, such as environmental protection agencies and climate scientists, may be interested in different properties of the soils. It is unrealistic to measure soil density, heavy metal concentration, and biological properties, such as bio-abundance and diversity from a single fixed installation. Third, soil properties are evolving phenomena and require continuous monitoring, which often requires a redeployment or a new deployment of sensors at different locations. Many planning-based sensor deployment methods also need user requirements such as coverage and spatial/temporal resolution of the sensed data. This is a “know-it-all” approach that is often not tenable for practical applications. For example, to maximize the yield of a new crop, agricultural specialists often gradually understand what soil condition to monitor and the proper temporal and spatial granularity. These challenges are compounded to form a significant obstacle to building low-cost and scalable soil monitoring systems that can adapt to changing soil conditions and to meet evolving user requirements.

Aiming to address these challenges and continuing on our prior work [6], we propose a soil monitoring framework that integrates predictive modeling, land-based in situ sensing, and mobile sensors based on autonomous robotic vehicles. The proposed approach complements traditional soil surveys by offering observations on specific quantities with higher spatial and temporal resolutions at selected locations.

At the core of the proposed framework is active learning-based predictive model building. The approach is to dynamically and gradually deploy sensors based on application requirements. The main idea is to iteratively determine sensor locations, in particular, the locations of mobile sensors, in parallel to the process from which the user acquires an understanding of their application requirements. The active-learning-based process removes the need to compute a fixed sensor development plan that is a hard computational problem (generally a Nondeterministic Polynomial- or NP-hard problem [11]). Further, it no longer requires full knowledge about the user’s application requirements on the outset.

We use autonomous robots for collecting data from environments. Due to recent progress in autonomy and data processing capability in Unmanned Ground Vehicles (UGV) and Unmanned Aerial Vehicles (UAVs) [12], these vehicles can be equipped with sensors to collect physical, chemical, and biological attributes to support a variety of users. Compared to fixed land-based sensors, the robotic platforms can provide extensive area coverage with a relatively small number of units. The use of mobile sensors enables the practicality of the active learning-based approach. UGVs/UAVs allows us to deploy sensors on demand and adjust to the modeling results of the monitored physical/chemical/biological attributes.

Our predictive models are built upon machine learning algorithms aided by the active-learning-based sensor Deployment. Complex physical/chemical laws govern soil modeling. It is difficult to predict/interpolate/extrapolate certain soil conditions that are not directly observed. Machine learning as a data-driven approach is capable of inferring a complex model from observed data alone. Our system uses machine learning to interpolate observed quantifies spatially and temporally, but more importantly, predict quantities that are not directly observed. For example, soil density and soil moisture can be predicted from multi-spectral image data observed via UAV [13,14]. As a result, the models provide high-accuracy data at locations where no sensors are present or certain quantities of interest where precise direct sensor observations are not available, which reduces required sensors and thus reduce operating and maintenance cost.

The contribution of this work is as follows:

1. We propose a heterogeneous sensing framework that features land-based stationary sensors, mobile sensors onboard UAVs and UGVs. This framework takes advantage of development in sensing technologies, sensor networks, and robotics and provides a sensing coverage aiming to meet the requirements of different users. We envision that a soil monitoring system designed in this framework will need fewer sensors than otherwise, which offers an advantage in operating and maintenance cost.
2. Modeling is an essential component of the design. The machine learning-based modeling interpolates and predicts quantities of interest from collected data. An important feature of the design is to reserve a set of sensors called the “calibration sensors”. These sensors are not used for building the machine learning models, rather, they are to check the interpolation and prediction accuracies, whose requirements are determined by the users.
3. In contrast to the conventional planning-based sensor development, our approach is characterized by an active learning-based sensor deployment. This approach is an iterative model building process. The process is integrated with a multi-robot planning algorithm, via active learning, the algorithm dispatches UAVs and UGVs with onboard sensors to collect quantities of interest to meet the users’ requirements on temporal and spatial resolution and accuracies of the predictive models.

## 2. Related Work

Several areas of research and development are a motivation and foundation of this work.

### 2.1. Soil Sensing

Modern soil sensors takes advantage of a variety of sensing mechanisms and offer in situ measurement of a range of soil properties. The sensing mechanisms range from electrical and electromagnetic, optical and radiometric, mechanical and electromechanical, acoustic, pneumatic, to electrochemical. The sensors can measure soil texture, density, carbon content, moisture, salinity, pH level (acidity/basicity), nitrogen content, and carbon exchange capacity [15]. In contrast, remote sensing measures reflected and emitted radiation of an area at a distance, typically via a satellite or an aircraft [16]. UAVs ease the acquisition of high resolution remote sensing images, such as multispectral images for soil monitoring, in particular, when we correlate the images with other soil properties [17].

This work neither directly contributes to the development of in situ and proximal soil sensors, nor to that of remote sensing technology. Instead, it proposes a framework to leverage this sensing technology to monitor soil conditions in a large terrain at scale for a range of users.

### 2.2. Digital Soil Mapping and Machine Learning

Soil surveys are a traditional method to create soil maps. However, the soil maps are static, with coarse spatial granularity, and often based on obsolete data [18]. Digital soil mapping (DSM) creates soil maps via computerized processing of in situ soil sensing, proximal soil sensing, and remote sensing data [18]. The advantages of DSM include the ability to update and reproduce soil maps frequently.

Another advantage of DSM is its potential to provide quantitative predictions of soil attributes along with uncertainty quantification and to avail an interpretation or a ranking of the importance of pedogenic factors on soil attribute distribution via computational and statistical models [18]. Researchers investigated a variety of empirical approaches for spatial predictions of soil attributes at unvisited locations, the locations where no direct observations are taken [19]. These prediction methods are generally a coarse approximation of the complex physical or chemical reality, e.g., assuming a linear model [19].

Recently there has been a growing interest in applying machine learning models to predict soil attributes [20]. Machine learning models can discover complex patterns and rules in the data and apply the patterns and rules to soil attribute prediction. A variety of machine learning models have been applied to soil attributes prediction, and these models include regressions (e.g., multiple linear regression), clustering (k-nearest neighbor), classification (support vector machine, random forest), neural network models [20]. There are a number of challenges in machine learning based models. One is the acquisition of sufficient “model-building” data, and the other is about evaluation, i.e., how we know the model can predict well for unknown attributes as such a model can overfit the training data, which is so-called the generalization problem.

With regard to the recent advance of using machine learning for predicting soil attributes, our approach is unique. First, current machine learning-based models assume the availability of large soil observation datasets as training data, which is often not realistic because the soil survey data are often static and expired. Prior models commonly use a cross-validation approach to evaluate the models, and do not take into consideration how the model should be evolved to future observations. Our framework is to take advantage of recent advances in soil sensing and unmanned robotics in order to provide sensing coverage and resolution at scale. More importantly, we propose an active learning-based modeling building and evaluation process [21,22]—as we shall discuss, using mobile sensors onboard unmanned vehicles, we can iteratively add sensing locations strategically in order to control the quality of predictive machine learning models. Combined with land-based sensors, our framework is aimed at effective evaluation and generalization of the model via a selected set of calibration sensors.

### 2.3. Path Planning for Mobile Robots

The proposed framework features a set of unmanned vehicles, or mobile robots to carry mobile sensors. Iteratively, the framework selects a list of sensing locations and dispatches the vehicles to collect sensor data. Carefully planned paths of mobile robots to visit these locations can speed up sensor data collection and reduce the cost of robot navigation. This problem is closely related to multi-Travelling-Salseman Problem (mTSP) [23], a known NP-hard problem. The goal of mTSP is to calculate the visiting sequence of sites for each robot while minimizing a cost function. Classical mTSP formulation assume a simple navigation cost between two connected nodes without concerning the physical obstacles.

For single robot path planning in environments with obstacles, the Probability Roadmap (PRM) algorithm [24] is widely used. The algorithm takes random samples of the map’s free space, testing them for their reachability, and use a local planner to connect these configurations to form a graph. A search algorithm is applied to the resulting graph to determine a path between the starting and goal configurations.

Multi-robot Information-driven Path Planning (MIPP) and other Gaussian Processes (GP) based approaches build a model of the spatial distribution of the phenomena of interest using mutual information entropy between different locations in the environment [25]. Robots are commanded towards high information entropy regions.

Another related area is the multi-robot Simultaneous Localization and Mapping (SLAM) problem [26,27,28]. There the objective is to stitch multiple local maps together to form a global map for path planning, where each local map is constructed using local range and/or vision sensors. Typical sensors for map building include LIDAR, stereo cameras, and even regular mono-color cameras. SLAM problems are often solved as non-linear optimization problems by minimizing the errors between the robot observation and a generative parametrized trajectory model. The execution of the multi-robot paths in our proposed framework is controlled by the robot SLAM and navigation software stack using the GTSAM implementation [28].

Our multi-robot path planning problem differs from the mTSP in several aspects. First, the cost model is different. The main component in our cost model is the projected accuracy of the underlying predictive model which is constructed by the visited sensor locations. As such, our path planning solution is a tour to a subset of sensor sites from a larger pool of candidates, but not the entire set of the candidates.

## 3. Model-Driven Sensing Framework

**Figure 1:**
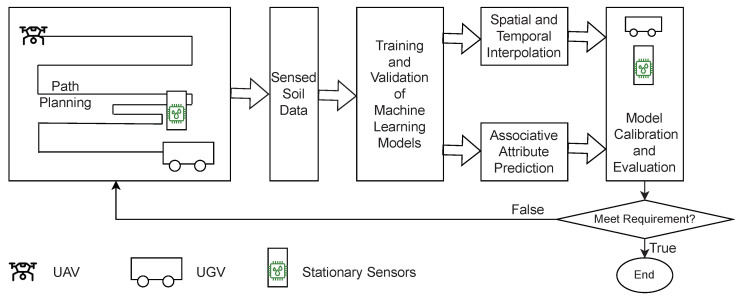
Model-driven sensing framework with heterogeneous sensor networks and active learning.

Our soil sensing framework integrates stationary and mobile sensors within a predictive modeling and machine learning computational paradigm. Figure 1 illustrates the proposed sensing framework. The framework features a heterogeneous sensor network, i.e., a sensor network consisting of stationary and mobile sensors. Stationary sensors are land-based fixed sensors while mobile sensors are mounted on unmanned robotic vehicles (e.g., UAVs and UGVs). Permanently installed soil sensor devices usually have multiple special sensors to detect a wide range of physical, chemical, and others properties of the soil. The robotic vehicles (UAVs or UGVs) can be fitted with compact sensors to collect data. Several commercially available robotic vehicles can be used, including the Clearpath Jackal robot that is a mobile robot of choice for numerous research projects and current in use in our lab [29,30] (The Clearpath Jackal robot is a product of Clearpath Robotics Inc., Ontario, Canada. For more information, see https://clearpathrobotics.com/jackal-small-unmanned-ground-vehicle/, accessed on 12 February 2023). The examples of the mobile sensors or sensors onboard robotic vehicles can be a gamma spectrometer, an electromagnetic induction instrument, and a ground facing camera [31]. Additionally, we assume that UAVs can collect remote sensing data as a secondary input. An example of the UAV sensor data is multi-spectrum imaging data, which can be used to predict the interested soil condition, such as soil moisture, soil carbon content, or soil bulk density [13,14,32,33,34].

The centerpiece of our framework is predictive modeling. Via the data collected from the heterogeneous sensor network, we can build two types of *predictive models*.

- Same attribute interpolation model (or interpolation model). From the collected sensor data, this model estimates the distribution of the observed attributes, which allow us to further predicts the same soil attributes at an arbitrary location or in a future time instance. We refer to the former as spatial interpolation and the later temporal interpolation. For brevity, we call this type of predictive model *interpolation model*.
- Associative attribute prediction model. This model is built from the data of a set of attributes; however, the model estimates one or more attributes that are different from the set of attributes. For instance, we can use multi-spectral images to build a model to estimate soil bulk density. For brevity, we call this type of predictive model *prediction model*.

There are several challenges to building these predictive models. Traditional predictive modeling requires a thorough understanding of the underlying physical/chemical processes in soils. In many cases, a precise model requires data on soil types, which can vary significantly by location. These imply that not only do we need domain knowledge/expertise in the physical and chemical processes, but also we often end up with different predictive models for different regions and parameters setting. To address these two challenges, to reduce the dependency on specific domain expertise and to obtain a more generic predictive modeling framework, we propose a data-driven approach and leverage machine learning algorithms to build predictive models.

Our approach is inspired by the recent success of machine learning algorithms in discovering complex pattern and latent associations in high dimensional datasets. Our work addresses the fundamental problem, i.e., whether the algorithms via the two types of predictive models can produce sufficiently accurate values of soil condition of interest at desired spatial and temporal resolution. The proposed active learning based model building pipeline adopts a unique treatment to process the sensor data in three distinct stages as shown in Figure 1:

- Initial training sensors (or fixed sensors). We plan an initial deployment of a few stationary sensor, and use the data collected from these initial set of sensors to build a machine learning-based model for interpolation and associative attribute prediction. These sensors are *fixed* because their deployment locations do not change being a mobile sensor or a stationary sensor.
- Calibration sensors. These are a set of sensors deployed to locations of the user’s interest to determine whether the framework’s model provides adequate temporal and spatial resolution and accuracy for the user’s application, such as monitoring soil condition for a particular agriculture crop. For instance, in our machine learning based predictive models, data collected via these sensors serve as “ground-truth” to estimate the accuracy of the models.
- Dynamically deployed sensors (or dynamic sensors). Based on the accuracy of the predictive models and the user’s requirement, we determine the next iteration of the active learning model building process. If the accuracy is not sufficient, continue the active learning process and select locations to set up stationary sensors or to dispatch mobile sensors. The data collected from these sensors in conjunction with those collected from the initial training sensors are used to build or improve the predictive models. For convenience, we call collectively the initial training sensors and the dynamic sensors as *training sensors*. The sensing locations of the training sensors gradually grow as the result of active learning.

Algorithm 1 illustrates the concept of this active learning model building pipeline. The algorithm first divides the initial batch of sensors into the training sensors ($T_{0}$) and calibration sensors ($C_{0}$). The sensor data ($D_{i}$) from the training sensors is used to construct a predictive model ($M_{i}$). Next, we evaluate whether the model meets the user requirements based on the ground-truth observation from the calibration sensors ($C_{i}$). If the model performance is less than satisfactory, the algorithm selects additional sets of training and calibration sensors and deployment locations ($S_{t,i}$, $S_{c,i}$). This is followed by a model updating using the new training and calibration sensor sets. The entire process will iterate until the model converge.
**Algorithm 1:** Active learning for building predictive models
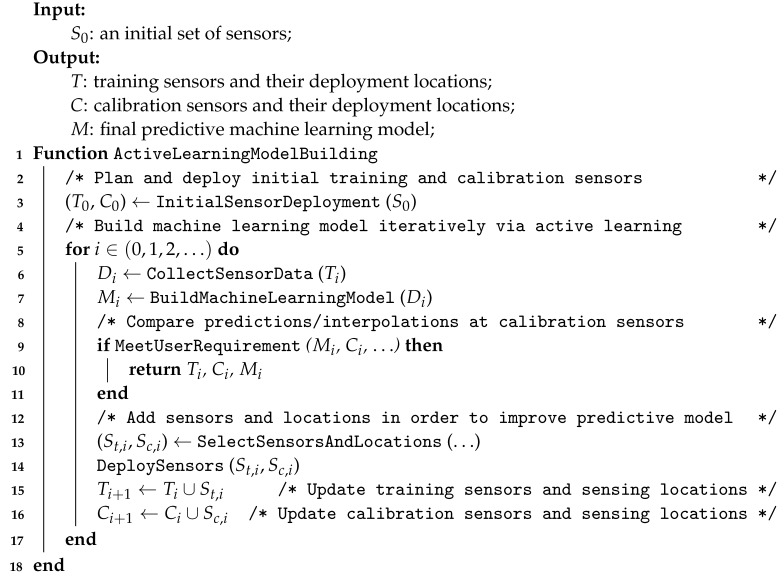


## 4. System Design

This section presents the main computational components of our active learning framework. Two machine learning models are used for model fitting: a spatial interpolation model and an associative attribute prediction model. Both models are based on the Gaussian process (Section 4.2). The two predictive machine learning models are embedded in the active learning process (Algorithm 2). A multi-robot sensing assignment and path planning algorithm is also discussed.

### 4.1. Predictive Models

Following a probabilistic machine learning approach, we consider two types of models as follows [35]:

- A spatial interpolation model assumes that the target function f(l)=q is a spatial function, where the input $l=(l_{x},l_{y},l_{z})$ specify a spatial location. In our case, similar to many GIS systems, we can use the geographical coordinate $\left(l_{x},l_{y}\right)$ and depth $l_{z}$ as input. The predicted quantity *q* can be any soil properties observed. Given a set of observations $Q=(q_{0},q_{1},q_{2},\ldots )$ observed at locations $L=(l_{0},l_{1},l_{2},\ldots )$, we can approximate the function *f* by fitting the function with the given data. The dataset will often contain noise, outliers, and are likely incomplete. There is uncertainty to determine what *f* is. To address the uncertainty, a probabilistic approach is to infer from the data a probabilistic distribution of function *f*, i.e., to infer p(f|Q,L). The spatial interpolation essentially estimates the distribution of the interested qualities ($q_{*}$) at a given location ($l_{*}$),
  (1) $$p\left(q_{*}|l_{*},Q,L\right)=\int p\left(q_{*}|f,l_{*}\right)p\left(f|Q,L\right)df$$
- A different predictive model f(o)=q assume the input *o* is the observations of a set of soil attributes. Given a set of observations $O=(o_{0},o_{1},o_{2},\ldots )$ and $Q=(q_{0},q_{1},q_{2},\ldots )$, we are to similarly determine function *f*. Likewise, the data can have noise, may not be complete, can also have errors. To make a prediction of $q_{*}$ for an observation $o_{*}$, we compute,
  (2) $$p\left(q_{*}|o_{*},O,Q\right)=\int p\left(q_{*}|f,o_{*}\right)p\left(f|O,Q\right)df$$
  There is flexibility about what constitutes an observation. To monitor soil condition, we typically treat $o_{i}=\left(q_{i,1},q_{i,2},\ldots ,q_{i,n_{q}},l_{i}\right)$ where $q_{i,1},q_{i,2},\ldots ,q_{i,n_{q}}$ are the values of $n_{q}$ attributes of interest, such as moisture, temperature, and others while $l_{i}=\left(l_{i,x},l_{i,y},l_{i,z}\right)$ is the location where we observe the attributes. However, we can also consider for some applications $o_{i}=\left(q_{i,1},q_{i,2},\ldots ,q_{i,n_{q}}\right)$ where location is irrelevant.

Observing the similarity between Equations (1) and (2), we unify the interpolation and the prediction models. We compute p(f|X,y) where *X* is the observed data on a set of “independent variables” and *y* is the dependent variable. To make a prediction on a new value of the independent variables ($x_{*}$), we will evaluate

(3) $$p\left(y_{*}|x_{*},X,y\right)=\int p\left(y_{*}|f,x_{*}\right)p\left(f|X,y\right)df$$

For the interpolation model, we let $x=l=(l_{x},l_{y},l_{z})$, the sensing location and *y*, a quantity we are interested in. For the prediction model, we set $x=o=(q_{1},q_{2},\ldots ,q_{n_{q}},l)$ where $\left(q_{1},q_{2},\ldots ,q_{n_{q}}\right)$ is $n_{q}$ quantities of interest and *l* the location. Alternative, for the prediction model, we let $x=o=(q_{1},q_{2},\ldots ,q_{n_{q}})$ when location is irrelevant. We solve the unified model via Gaussian Process.
**Algorithm 2:** Model-based heterogeneous sensing with active learning and Gaussian process
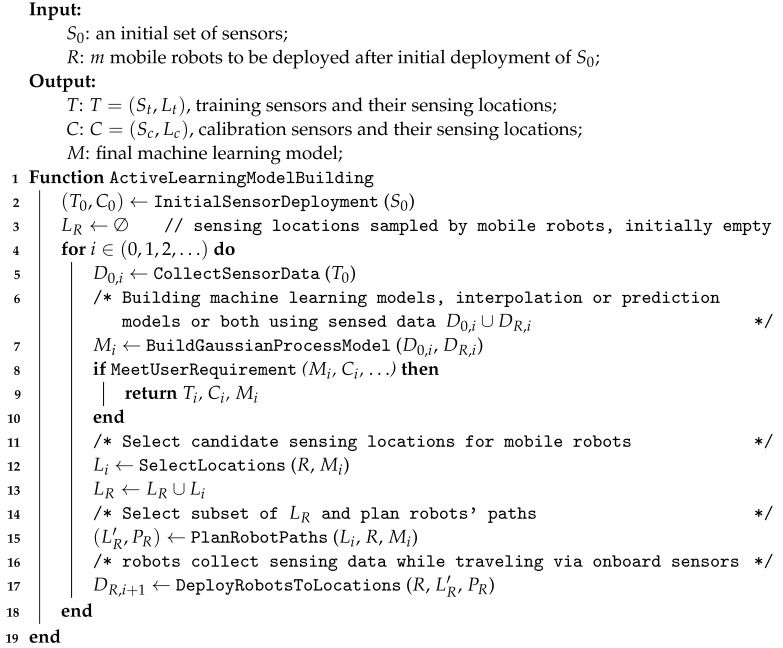


### 4.2. Gaussian Process Models

The probabilistic models in Section 4.1 require us to compute a probability distribution of function in the general case, i.e., p(f|X,y). An efficient method to define and calculate such a distribution is via Gaussian process (GP) [35]. The general form of GP is defined as $p\left(y_{1},y_{2},\ldots ,y_{n}\right)=p\left(f\left(x_{1}\right),f\left(x_{2}\right),\ldots ,f\left(x_{n}\right)\right)$, a distribution over the values of the function at a finite, but arbitrary set of points, $x_{1},x_{2},\ldots ,x_{n}$ where $f\left(x_{i}\right)=y_{i}$, i=1,2,…,n. In GP models, we assume $p(f\left(x_{1}\right),f\left(x_{2}\right),\ldots ,f\left(x_{n}\right))$ is a Gaussian distribution whose mean is μ(x) and covariance Σ(x) where $x=(x_{1},x_{2},\ldots ,x_{n})$. Covariance Σ(x) is given by a covariance function, calculated by a kernel function, i.e., $\Sigma_{ij}=\Sigma \left(x_{i},x_{j}\right)=\kappa \left(x_{i},x_{j}\right)$.

The intuition behind GP is to predict an unknow datapoint based on its similarity to the known or trained observations. Simply put, given an input, say $x_{*}$, the machine learning model predict an output, say $y_{*}=f\left(x_{*}\right)$ to be similar to the data, e.g., if $x_{i}$ and $x_{j}$ are similar to $x_{*}$, then the model should be made such that $f(x_{*})$ is similar to $f(x_{i})$ and $f(x_{j})$. The covariance function $\kappa (x_{i},x_{j})$ can be thought of as a measure of “similarity” between points $x_{k}$, k=1,2,…,n. For a given point $x_{*}$ whose function value is sought for, if $x_{*}$ is similar to $x_{i}$ and $x_{j}$, $y_{*}=f\left(x_{*}\right)$ should be similar to $y_{i}=f\left(x_{i}\right)$ and $y_{j}=f\left(x_{j}\right)$ [35]. Because of this, the success of a Gaussian process model is by and large determined by the kernel function that encodes prior knowledge about the similarity of inputs observations $x_{i}$.

The literature has investigated a number of kernel functions for covariance Σ(x) [36]. Some of these kernel functions, such as the Radial Basis Function (RBF) kernel, are stationary while the others not, such as the dot product kernel. Some are very smooth (infinitely differentiable), such as RBF and the others rough, such as the Matérn kernels. Kernel functions can also be periodic, such as the exp-sine-squared kernel or aperiodic. Experimenting with different kernels is a method to select the most appropriate one for the data in hand or the quantities of interest.

Gaussian process models have the advantage that we obtain the probabilistic distribution of $y_{*}$ give $x_{*}$. Since marginalization, summations, and conditioning of a Gaussian distribution remains a Gaussian distribution, following the Gaussian assumption (discussed above), $p(y_{*}|x_{*},X,y)$ is a Gaussian distribution that we fully characterize by its mean and variance [35,37]. Given this, not only can we obtain a point estimate about $y_{*}$, such as the mean (i.e., ${\bar{y}}_{*}$), but also the uncertainty of the point estimate. In Section 4.3.2 we shall discuss how we take advantage of this to select mobile sensor locations to complete the active learning model building process.

### 4.3. Mobile Robot Sensing and Path Planning

After an initial deployment of sensors, we leverage *m* mobile robots to visit to perform in situ sensing (or proximal sensing) at a set of locations. The overall objective is to identify *p* best sensing locations for the *m* robots to visit in order to improve sensing resolution and predictive models’ accuracies. The problem can be loosely defined as the multi-Travelling Salesman Problem (mTSP) [23], albeit with a different model of path cost. Our multi-robot path planning problem at hand has the following difference over the classical mTSP problem setting:

- The set of locations where robots must visit and perform sensing is not pre-determined. Rather, a set of candidate locations is derived from the probabilistic model built from the sensor data already collected (Section 4.3.2). Our algorithm must select a set of new locations for new sensor data. The choice of locations must minimize the errors of the updated data model once the new sensor data is collected by the mobile robots.
- The mobile robot path planning algorithm minimizes a cost/objective function that contains two parts: (1) the expected errors of the predictive models over the survey area post deployment of the mobile robots, and (2) the total time required for the robots to complete the survey mission. The second part is similar to the cost function in a classical mTSP problem setting [23]. However, in our work, the two cost terms should be weighted in the overall cost function.

The path planning problem is a non-linear optimization problem. Given the path cost function, the problem is not “easier” than mTSP. The mTSP problem is NP-hard and are typically solved by heuristic algorithms [23]. In this work, we focused on a simple greedy approximation by decomposing the problem into two steps:

1. Find the *p* data locations to dispatch the mobile robots, with the objective to minimize the modeling error;
2. Run an mTSP algorithm to minimize the robot navigation cost.

**Figure 2:**
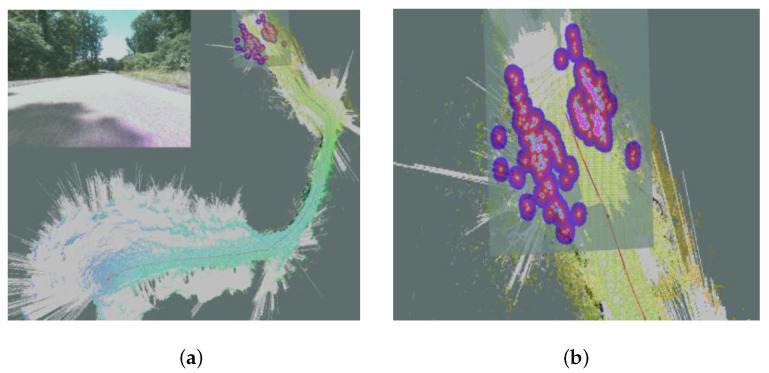
This is the global and local maps constructed by an autonomous robot when exploring an unknown terrain. The gray area represents the unknown area. (**a**) Robot autonomous navigation and mapping; (**b**) local map with observed obstacles.

Additionally, we also consider the following:

- We assume a uniform terrain model such that the navigation cost is a linear function of the Euclidean distance (i.e., L2 distance) traveled. In practice, navigation cost by itself can be modeled from robots’ deployments, in particular, implemented in conjunction with SLAM. Figure 2a shows the map built by a robot when it explores an unknown area.
- The robot navigation can be realized using either a centralized solution or a distributed solution. A distributed solution could use an auction-bidding process where a moderator will issue next locations to visit, and each robot bid on the next available target locations. The bid is the Euclidean distance from the robot’s current location to the goal, the lowest bid wins, and the robot selects that goal. When there is a structural change to the communications network, meaning a robot is added or removed, all auctions are restarted, with the lowest bid winning. Any robot that loses the auction returns to the start of the process by waiting for a new auction to win before starting their own.

#### 4.3.1. Path Planning

The candidate sensing locations and possible paths are represented by a complete graph G=(V,E). The vertex or node set *V* to represent the $n_{v}$ candidate locations while *E* is the candidate paths. From *V* we select *K*, $p\leq K\leq n_{v}$ locations that forms a connected component of *G*. For each edge, the associated navigation cost is denoted as $c_{i}$, $i=0,1,2,\ldots c_{n_{e}}$. The set of robots is denoted as $R=r_{i},i=1,2,\ldots ,m$. Binary variable ${\left\{l\right\}}_{i,j}\in \left\{0,1\right\}$ is set to 1 if a location $v_{i}\in V$ is assigned to robot $r_{j}$. Binary variable ${\left\{e\right\}}_{i,j}\in \left\{0,1\right\}$ is 1 if edge $e_{i}$ is assigned to robot $r_{j}$. We aim to minimize the cost function *c*:

(4) $$c=w_{1}\sum_{v_{i}\in V,r_{j}\in R}||g\left(v_{i}\right)-f\left(v_{i}\right)\left|\right|{\left\{l\right\}}_{i,j}+w_{2}\sum_{i\in R,j\in E}c_{i}{\left\{e\right\}}_{i,j}$$

with constraints

(5) $${\left\{l\right\}}_{1,i}=1,r_{i}\in R$$

(6) $$\cup \{e_{i}|{\left\{e\right\}}_{i,j}=1,e_{i}\in E,r_{j}\in R\}\;\mathrm{contains}\;a\;\mathrm{Hamiltonian}\;\mathrm{path}$$

(7) $${\left\{e\right\}}_{i,j}=0,i\in \left\{i_{1},i_{2}\right\},l_{i_{1},j}=0,l_{i_{2},j}=0,e_{i}\in E,r_{j}\in R$$

(8) $$\sum_{v_{i}\in V,r_{j}\in R}{\left\{l\right\}}_{i,j}\geq p$$

(9) $$\sum_{v_{i_{1}}\in V}{\left\{l\right\}}_{i_{1},j}=\sum_{v_{i_{2}}\in V}{\left\{l\right\}}_{i_{2},j},r_{j}\in R$$

Cost function (4) has two terms. The first is the modeling error and the second is the robot navigation cost where $w_{1}$ and $w_{2}$ are two weights, and $w_{1}+w_{2}=1$, $0\leq w_{1}\leq 1$, and $0\leq w_{2}\leq 1$. Function g(·) is the ground truth of the data field, and function f(·) represents the predicted data field with the addition of the mobile robots. The error term of the data field is thus denoted by $w_{1}\sum_{v_{i}\in V,r_{j}\in R}||g\left(v_{i}\right)-f\left(v_{i}\right)\left|\right|{\left\{l\right\}}_{i,j}$. The navigation cost becomes $w_{2}\sum_{i\in R,j\in E}c_{i}{\left\{e\right\}}_{i,j}$ the sum of path costs of the robots weighted by $w_{2}$. If K=p and $w_{1}=0$, the problem becomes the classical mTSP problem and can be solved as an instance of Mixed Integer Linear Programming (MILP), whose solver is available in many optimization packages.

Constraint by Equation (5) is to ensure all robots begin from “home”, i.e., depart from node 1, the starting location. Equation (6) enforces that the assigned location to any robot must form a path, which is feasible since we assume the graph is complete and any two nodes are reachable from each other. Equation (7) requires that an edge cannot be assigned to a robot if none of its incidental vertices are assigned to the robot. Next, Equation (8) reflects the requirement that we must schedule the robots to visit at least *p* locations. Finally, Equation (9) must hold because the locations visited by a robot becomes a path (i.e., the edges are connected).

#### 4.3.2. Selections of Sensing Locations

The predictive models are built iteratively. For each iterator, we select $n_{v}$ candidate locations to deploy *m* mobile sensors in order to improve the quality of the predictive models. This process is part of active learning model building in the proposed framework (Section 3).

There are a number of methods to determine the $n_{v}$ locations, for instance, based on mutual information entropy or based on prediction error. The intuition of the former comes from information theory. The locations to be sampled should favor areas with low information (high entropy), which is more useful to provide correction on the current predictive model. That of the latter is the result of probabilistic modeling. The probabilistic model yields a distribution of function *f* (Section 4.1) from a given set of observations, from which, we can also know the error distribution over the sensing area. We expect that adding sensing data from those locations where the errors are the greatest can reduce the errors.

For either, we propose a simple greedy algorithm to select the candidate data locations. The initial list of candidate points is selected at the grid points over the target domain D. We then calculate the local entropy value or standard errors on the initial list. We then select the top P candidate node. We can then select the top $n_{v}$ candidate node, such as using Non-Maximum-Suppression (NMS) [38]. The purpose of NMS is to prevent over-representing of some target areas with high entropy index or high error index. Algorithm 3 is the procedure to select the candidate locations via prediction error.
**Algorithm 3:** Sensing locations selection via standard error
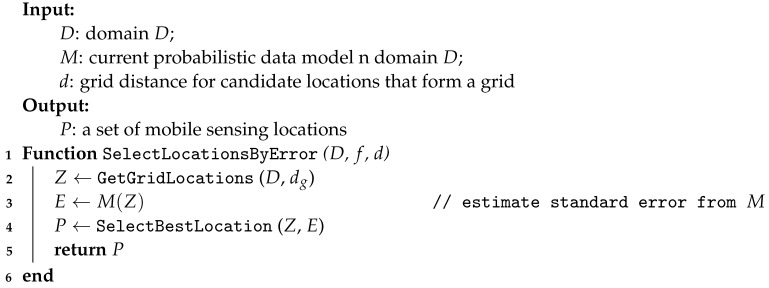


#### 4.3.3. Implementing Path Planning

The path planning formulation in Section 4.2 has a simplified navigation cost assuming a flat field with no terrain obstacles. In the real world, the actual navigation cost between two locations are more than the simple L2 distance of the two points. The real-world scenarios often have natural rock blocks or man-made obstacles between sensor locations; hence, the straight line is not necessarily the lowest-cost path or a feasible one due to the robot’s maximum climbing capability. To estimate the actual cost of a path, detailed and accurate altitude information of the target area is required. This information can be obtained from public datasets such as the US topographic map database [39], which also contain information on man-made and natural features on the ground, such as roads, railways, contours, elevations, and rivers. With the altitude information, the lowest path and its cost can be estimated using the probabilistic roadmap (PRM) algorithm  [24]. Given two sensing locations, the PRM takes random samples from a surrounding convex space, testing them for whether they are in the free space, and attempt to connect these configurations to other configurations to form a connected graph where each edge cost approximates the terrain-dependent cost. A graph search algorithm is then applied to determine the shortest path between the source and the destination. This computation only needs to be carried out once and the results are used by the UpdatePathCosts() function in the main path planning algorithm (Algorithm 4).
**Algorithm 4:** M-robot path planning
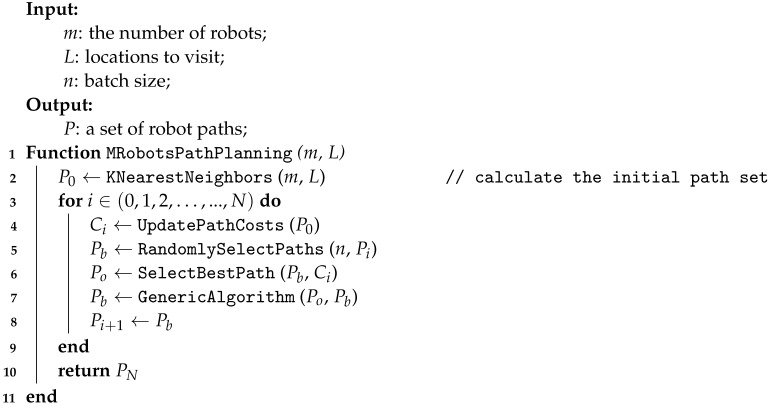


Next, we use a Genetic Algorithm (GA) based implementation [40] to generate the complete tour path for each robot. The algorithm uses a multi-chromosome genetic representation to code solutions into individuals. Each chromosome represents a sub-path of a candidate path. Special genetic operators, such as crossover, sliding, and flip are used to allow sub-path to be mutated and exchanged among the solution population. The main difference in our implementation is: (1) the number of salesmen (i.e., the number of robots in our case) is no longer an optimization goal and is treated as a constraint, (2) we use K-Nearest-Neighbor (KNN) clustering to generate the initial site allocation in the population, (3) the GA minimizes the max cost of the M robot, instead of the total cost in the original version. The algorithm is described in Algorithm 4 below.

## 5. Evaluation and Numerical Experiments

This section provides the assessment process for the quality of the Gaussian process models, the effectiveness of active learning, and the robot path planning algorithm. It is notable that the main focus of this work is the data processing framework on an established dataset. Nevertheless, it is necessary to discuss the characteristic of the robot and appropriate sensors/equipment in a deployed heterogeneous system. Table 1 summarizes the robot and sensor details.

The robot platform is model Jackal, a skid-driving autonomous robot manufactured by Clearpath Robotics. The Jackal robot is equipped with a suite of navigational sensors, including a LIDAR sensor, IMU sensor, and an accessory vision sensor to provide basic autonomous driving functionality. The navigational sensors allow the robot to plan a motion path toward a goal location. The built-in SLAM software stack can also avoid obstacles in the planned route and make local adjustments. The robot baseline configuration has an endurance of 4 h running at 2 m/s.

In a physical deployment, the field soil data are acquired using the in situ sensor or laboratory analysis of the soil sample collected by the robot. As shown in Table 1, many soil data can be collected by the robot in the field using the rugged sensor packaging. Soil moisture, canopy radiometer, salinity, pH, and nitrogen sensors are examples of in-site devices. For the type of data that cannot be measured with portable sensors, such as the metal concentrations, samples will be collected by the Jackal robot for lab test.

### 5.1. Evaluation Data Set

We use the Meuse data set for the evaluation and experiments [49,50]. The Meuse data set is publicly available [51,52] and is widely studied for soil study [53,54,55]. The dataset contains 155 soil samples collected in a floodplain of the Meuse river near Stein, South Limburg, in the Netherlands [56]. The main attributes include the concentrations (measured in parts-per-million or ppm) of four heavy metals: cadmium, copper, lead, and zinc. Additional attributes include the geolocations of the soil samples, the distances from the sampling points to the Meuse river, the flooding frequency classes (1 for high flooding frequency, 2 for medium flooding frequency, and 3 for no flooding), the types of soils following the Dutch soil classification system (1 for Rd10A—Calcareous weakly-developed meadow soils, 2 for Rd90C-VIII—Non-calcareous weakly-developed meadow soils, and 3 for Rd10C—Red Brick soil, fine-sandy, silty light clay). The elevation is measured as relative elevation above the local river bed in meters. The tabular data also record the percentage of organic matter, the presence of lime (0 for absent, 1 for present), and land use class (such as Aa for Agriculture but unspecified, Ab for Agriculture—sugar beets, Ag for Agriculture—small gains, and so on, in total, 16 classes). To help interpret the evaluation results below, we present descriptive statistics including minimum, maximum, mean, median, standard deviation of the concentrations of the 4 heavy metals in Table 2.

### 5.2. Evaluation Metrics

To evaluate the predictive models f:X↦y, where $X\in R^{d}$, d∈N, and y∈R, we choose two regression evaluation metrics, the R-squared ($R^{2}$) and the Mean-Absolute-Error (MAE) [57]. The R-squared is also called the coefficient of determination and is defined as $R^{2}=\sum \left(y_{*}-\bar{y}\right)/\sum \left(y-\bar{y}\right)$. It is “a standardized measure of how much of the variance in the dependent variable is ‘explained’ by the independent variables in the regression model [58].” High $R^{2}$ values indicate a good agreement between the regression model and the training data set [58,59]. Although $R^{2}$ is the de facto tool to evaluate regression models, there are caveats [58,59]. To supplement $R^{2}$, we also examine MAE, defined as $|y_{*}-y|$, the magnitude of error of the estimate when compared to the ground truth [57].

### 5.3. Predictive Models

We consider three predictive models, a *spatial interpolation* model that interpolates heavy metal concentrations, an associative attribute *prediction model* that predicts heavy metal concentrations at arbitrary locations, a *combined model* that integrates both interpolations and prediction. The building block of our predictive models is a Gaussian process model [60] with a Rational Quadratic kernel function $k\left(x_{i},x_{j}\right)={\left(1+d{\left(x_{i},x_{j}\right)}^{2}/\left(2\alpha l^{2}\right)\right)}^{-\alpha }$. Here d(·,·) is the Euclidean distance and is particularly suitable for spatial interpolation and prediction models where location is an independent variable. The kernel function is parameterized by α, the scale mixture parameter and *l*, the length scale of the kernel. Via a simple grid search, we set these two hyperparameters as α=10 and l=10 unless otherwise specified. During training, the injection noise is controlled by a hyperparameter $\alpha_{gp}$ added to the diagonal of the kernel matrix. This can be interpreted as the variance of additional Gaussian measurement noise on the training observations. We set it as $\alpha_{gp}=0.2$ unless otherwise specified.

In statistics and machine learning, one confounding problem is the trade-off between two types of error: bias errors and variance [61]. The former is the difference between a model’s prediction and the ground truth, while the latter is the spread of the model’s prediction on the data. A high bias error is often the result of under-fitting, which leads to inaccurate prediction. On the contrary, a high variance error means that the model does not generalize well to the extended dataset domain, which can result from overfitting. It is well-known that we cannot simultaneously reduce both types of error and a trade-off is often made for a particular modeling problem.

We use a 10-times 5-fold cross-validation technique to balance the trade-off between $R^{2}$ and MAE error. In the 5-fold step, we split the data into five equal-size partitions. For the *j*-th run, 1≤j≤5, we use 80% of the samples for training (partitions {i|1≤i≤5,i≠j}), and the rest 20% (partition *j*) for evaluating. We repeat the above 5-fold cross-validation ten times, each after randomly shuffling the samples. In the training data, the heavy metal concentrations are known. In the evaluation data, the heavy metal concentrations are treated as unknown during prediction and the prediction model’s outputs are $y_{*}$. By comparing the ground truth values of the evaluation data with the predicted, we compute two evaluation metrics, $R^{2}$ and MAE. Since we repeat the evaluation for 10×5=50 times, the two metrics are averaged over the 50 repetitions. The averaging can reduce variation in the model assessment, i.e., we can reproduce the metrics across multiple runs of the 10-times 5-fold cross validation despite randomness in the order of the data samples and the training procedure.

### 5.4. Baseline Predictive Model

For the spatial interpolation model, the locations ($l_{x}$ and $l_{y}$) of 80% of samples are for building the model, we then use the model to compute interpolations at the locations of the samples in the evaluation data. By comparing the interpolations with the group truth values, we compute two metrics, $R^{2}$ and MAE. The reported metrics in Table 3 are the averages of the two metrics and their standard deviation over the 50 repetitions.

Next, we build a prediction model to predict a new attribute from sensor data. A sensor here collects or computes four attributes, the distance to the Meuse river, the flood frequency class, the elevation, and the percentage of organic matter in soil. These attributes are independent variables. From a new observation, we want to predict a heavy metal concentration. Table 4 summarizes the predictive performance of the model.

Finally, we combine the interpolation and the prediction models. This is to treat the locations and the sensed data (e.g., the distance to the Meuse river, and the flood frequency class, the elevation, and the percentage of organic matter in soil) as independent variables, and predict the heavy metal concentrations. Table 5 gives $R^{2}$ and MAE for the model.

The experiment results are summarized in Table 3, Table 4 and Table 5. We have several observations:

- All three predictive models can fit training data very well.
- The prediction model and the combined model outperform the interpolation model in both $R^{2}$ and MAE by a wide margin.
- The performance metrics obtained from the evaluation data are not as good as those from training data. As such, the metrics from the training data alone cannot be used to determine the quality of the fit. Thus, it is important to use a separate data set to evaluate the model and to determine if the model meets the users’ need. This provides a justification of the calibration sensors for the proposed framework and system.
- The combined model can have a significant improvement over the standalone spatial interpolation model and the standalone prediction model. As shown, although the metrics are almost identical among Cadmium, Copper, and Zinc, the metrics of Lead is a significant improvement, namely, $R^{2}$ of the combined model is improved by (0.67−0.47)/0.47≈43% over the interpolation model and (0.67−0.59)/0.59≈14% over the standalone prediction model.

### 5.5. Predictive Model via Active Learning

This section examines the predictive modeling augmented with active learning strategy. We set m=1 and fix to a single location to deploy the robot to collect samples.

We re-examine three models, a spatial interpolation, a prediction, and a combined model in numerical experiments. With active learning strategy, we divide the data samples into three parts, the evaluation data set, the initial training set, and the candidate data set for improving the predictive models. The evaluation data set consists of randomly selected 20% samples from the whole data set. The initial training data set is 8% random samples, i.e., 10% randomly sampled from the remaining 80% samples. The candidate data set is the rest, 72% random samples whose locations are to be visited by a mobile robot. The active learning model building process follows Algorithm 2 where it selects new sensing location via Algorithm 3.

**Figure 3:**
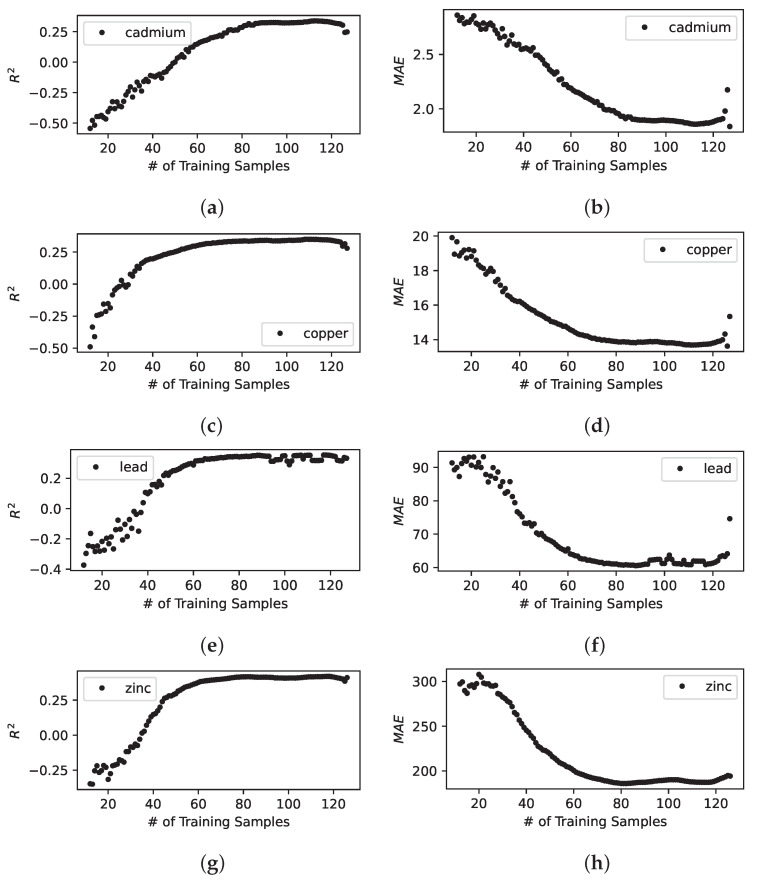
Standalone spatial interpolation model built with active learning. The figures show $R^{2}$ and MAE for predicted heavy metal concentrations on the evaluation data set. (**a**) $R^{2}$ for interpolated cadmium concentration; (**b**) MAE for interpolated cadmium concentration; (**c**) $R^{2}$ for interpolated copper concentration; (**d**) MAE for interpolated copper concentration; (**e**) $R^{2}$ for interpolated lead concentration; (**f**) MAE for interpolated lead concentration; (**g**) $R^{2}$ for interpolated zinc concentration; (**h**) MAE for interpolated zinc concentration.

**Figure 4:**
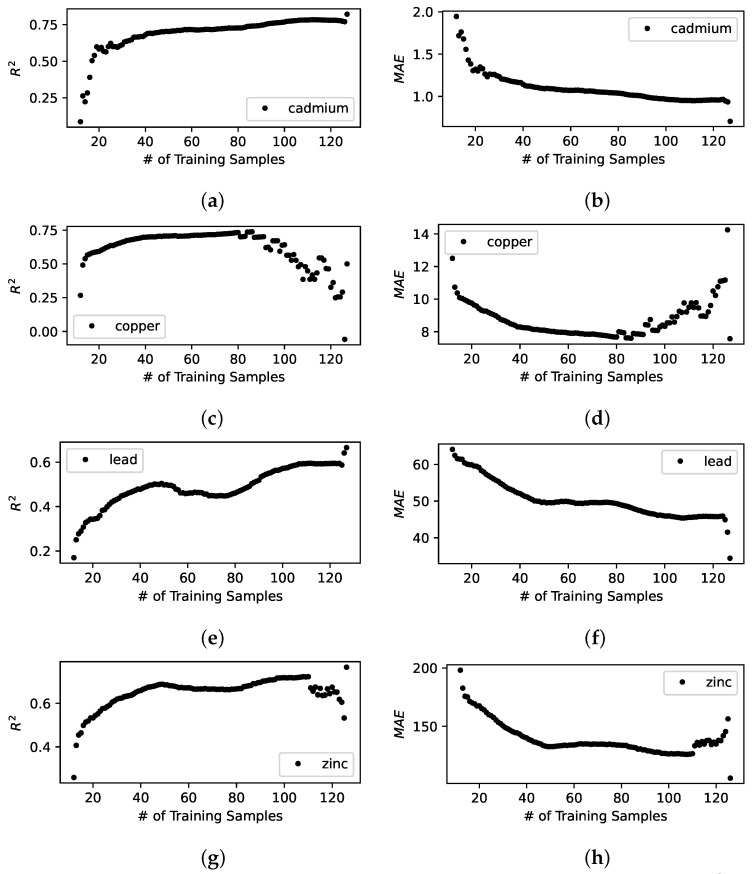
Standalone prediction model built with active learning. The figures show $R^{2}$ and MAE for predicted heavy metal concentrations on the evaluation data set. (**a**) $R^{2}$ for predicted cadmium concentration; (**b**) MAE for predicted cadmium concentration; (**c**) $R^{2}$ for predicted copper concentration; (**d**) MAE for predicted copper concentration; (**e**) $R^{2}$ for predicted lead concentration; (**f**) MAE for predicted lead concentration; (**g**) $R^{2}$ for predicted zinc concentration; (**h**) MAE for predicted zinc concentration.

**Figure 5:**
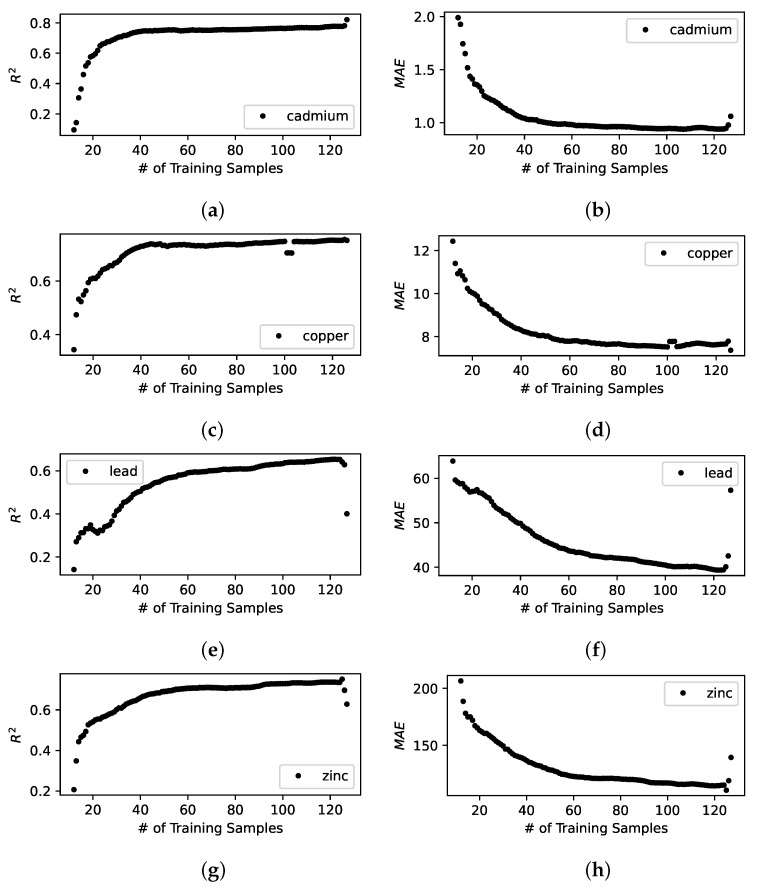
Combined interpolation and prediction model built with active learning. The figures show $R^{2}$ and MAE for predicted heavy metal concentrations on the evaluation data set. (**a**) $R^{2}$ for predicted cadmium concentration; (**b**) MAE for predicted cadmium concentration; (**c**) $R^{2}$ for predicted copper concentration; (**d**) MAE for predicted copper concentration; (**e**) $R^{2}$ for predicted lead concentration; (**f**) MAE for predicted lead concentration; (**g**) $R^{2}$ for predicted zinc concentration; (**h**) MAE for predicted zinc concentration.

Figure 3, Figure 4 and Figure 5 illustrates the model building process of the spatial interpolation model, the prediction model, and the combined model. We observe the following:

1. These results shows that $R^{2}$ and MAE improves quickly as new sensing locations are visited by a mobile sensor over iterations.
2. Once the model converges to the best predictive performance indicated by the greatest $R^{2}$ and the lowest MAE; however, it does not help further by adding more sensing locations and will eventually degrade the predictive performance. This is exactly what we would expect from an effective active learning model building pipeline that aims to select the best sensing locations at each iteration—since we evaluate the model by using a fixed set of candidate sensing locations, eventually, we are left with a set of sensing locations that are or whose data samples are out of distribution, such as less informing, or more noisy, or erroneous, or lack of constraining sensors nearby.
3. The combined model significantly improves not only the predictive performance but the stability or the trend of convergence of the model when compared to the other two.

### 5.6. Active Learning with Mobile Sensors (Robots)

**Figure 6:**
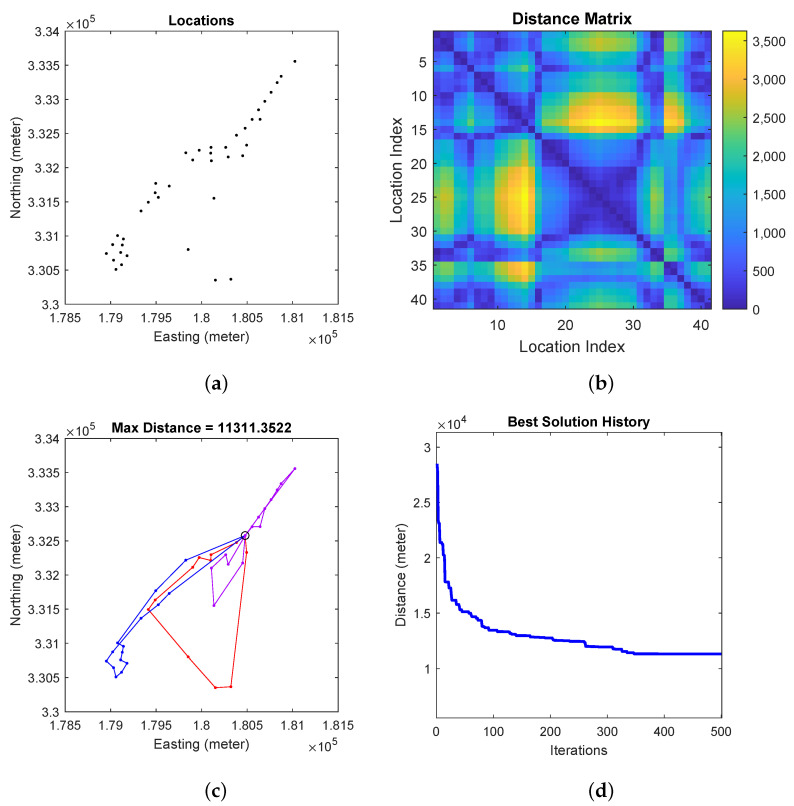
Generating robot paths for m=3 robots. (**a**) Sensing locations to be visited by the robots; (**b**) Distance matrix between any two sensing locations where a location is represented by its index. (**c**) Paths traveled by the m=3 robots when the total distance is minimized. We differentiate the paths of the m=3 robots using three different colors. (**d**) Total distance history over path planning iterations.

Finally, we present the numerical experiment with both active learning and multi-robot planning enabled. For mobile robot path planning. Figure 6 shows the generated location assignment and the corresponding visitation sequence for the robots. We fix m=3 for a three robot team. The resulting task requires each robot to travel about 14,000 m. As shown, the assigned workload of the three robot is balanced.

**Figure 7:**
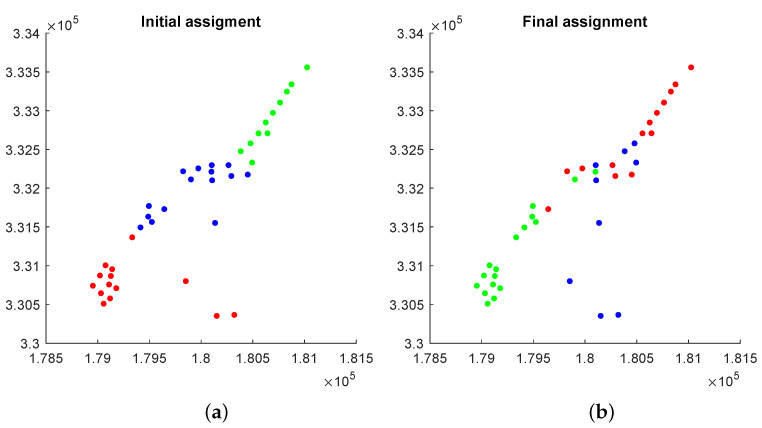
Sensing location assignments. We use three colors to differentiate assignments to the m=3 robots. (**a**) Initial sensing location assignment; (**b**) Final sensing location assignment.

**Figure 8:**
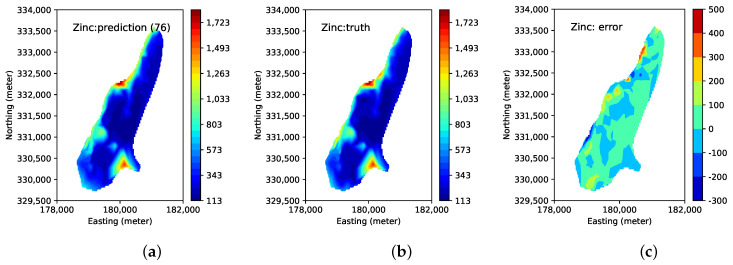
Predicted heavy concentration using the combined model, ground truth, and prediction error (i.e., $y_{*}-y$). (**a**): predicted Zinc concentration in soil; (**b**): ground truth of Zinc concentration; and (**c**): prediction error of Zinc concentration.

During the path planning process, the target sensing locations for each robot evolve by mutating, crossing-over, and shrinking within the feasible operations. Figure 7 shows the initial sensing locations and the final sensing locations when the model reaches an adequate performance requirement. In this case, the search will terminate when $R^{2}$ computed from the calibration sensors converge. Figure 8 shows the visualization of Zinc concentration built by the final predictive model. The ground truth and the sample locations are also shown. We select the best predictive model based on $R^{2}$ computed on the calibration sensors, i.e., we continue the active learning process until $R^{2}$ begins to drop after reaching the maximum, and select the model when $R^{2}$ is the greatest. We use the final model to predict the entire set of the Meuse data points (155 predicted values (denoted as $y_{*}$)). We also compute the prediction error (i.e., $y_{*}-y$) for the entire dataset. The result shows that the predicted concentrations and the ground truths are very close.

## 6. Discussion

We shall discuss some limitations of this work and future work that might address the limitations.

### 6.1. Bias-Variance Trade Off and Calibration Sensors

**Figure 9:**
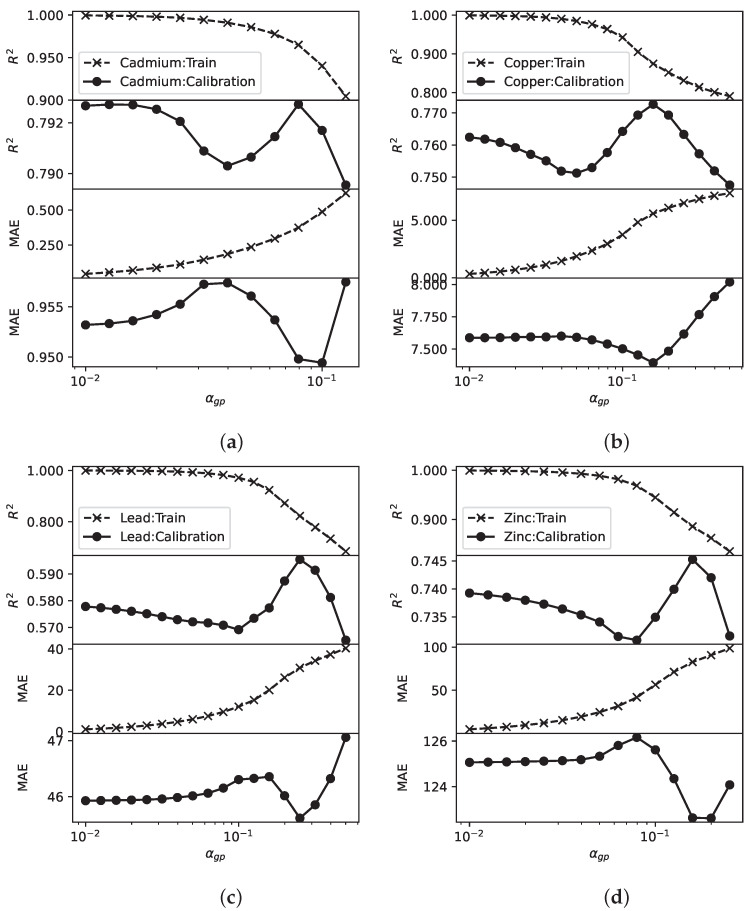
Bias-variance trade off. (**a**): Cademium; (**b**): Copper; (**c**): lead; (**d**): Zinc.

It is a common practice to evaluate a machine learning model using cross validation where we divide the data into training and test data. This concept motivates us to design our active learning model building with a set of calibration sensors. During active learning, we select a new sensing location based on a metric on predictive performance, e.g., in Algorithm 3, we select sensing locations with large predicted standard error. For this, we cannot rely on training data alone. We illustrate this in Figure 9. For this, our experimental setting is as follows. We conduct a 10 times 5-fold cross validation. The validation data is akin to the data collected by the calibration sensors. In the Gaussian process, we use hyperparameter $\alpha_{gp}$ to indicate the noise level in the data. The noise level is unknown and $\alpha_{gp}$ can be set to different values. Figure 9 shows that when $\alpha_{gp}$ is small, the Gaussian process model attempts to fit the data closely, which results in $R^{2}$ close to 1 and also low MAE. As $\alpha_{gp}$ increases, the model do not fit the data exactly, leading to smaller $R^{2}$. Without the evaluation data, the data from the calibration sensors, we would select models with high $R^{2}$ or low MAE. However, this is clearly problematic. $R^{2}$ obtained from the evaluation data will increase with $\alpha_{gp}$, an opposite trend to that obtained from training data. Thus, we should select sensing locations based on evaluation data.

### 6.2. Limitation and Threats to Validity

This work focuses on proposing a model building framework for soil sensing aimed to satisfy the application requirements of the users, such as agricultural specialist and climate scientists. To support the argument that the proposed framework can deliver soil data to the users in a cost-efficient fashion, we design a soil sensing system in the framework. The centerpiece of the system is the building of Gaussian process predictive models iteratively via active learning.

To evaluate the system, we conduct numerical experiments. We select two regression model performance metrics, $R^{2}$ and MAE. Users can have a diverse range of requirements, many of which are beyond our knowledge. Nevertheless, the end users may opt for different metrics to gauge the model building process.

For the evaluation, we must have the ground truth soil data. In this work, we leverage the Meuse data set available in the public domain. Clearly, the Meuse data set cannot represent soil conditions that exhibit far greater heterogeneity. As such, there is an internal thread of validity whether our observations obtained from the numerical experiments can hold for other data set.

The system relies on the Gaussian process models. With a Gaussian process model, we obtain in essence a joint probability distribution of the relevant variables and from the distribution, we can compute the variance and standard error. Because of this convenience, we choose to implement the active learning modeling building process leveraging the Gaussian process models. One limitation is that Gaussian process models can suffer from scalability problems when dealing with a large amount of data [62].

Additionally, this work gives emphasis to the model building process other than the best performing models. Recently deep learning models show promise for a wide range of applications [63]. It can be the case that deep learning-based models outperform the Gaussian process models examined in this work.

Last but not least, our numerical experiments assume a uniform terrain model such that the navigation cost is a linear function of the Euclidean distance traveled. This is an oversimplification, in particular, for ground mobile robots since the robots’ navigation cost depends on not only the distance traveled but also the terrain and the ground condition. The results of our navigation model can be extended to include terrain dependent costs as discussed in Section 4.3.3.

### 6.3. Future Work

To address the limitations, we consider four directions. First is to implement the active learning model building process with the mobile robot platforms we have and to deploy and experiment them in an agricultural field. This achieves two objectives. This shall be a usable system for agriculture users, and we shall collect additional sets of soil data samples to validate this study. Second is to extend this work to deep learning and other models, such as deep neural networks, ensemble models, and tree-based models [64,65]. We anticipate these models have the ability to capture complex soil conditions, and thus lead to superior predictive performance. The third is to integrate geographical features, chemical, physical, and geological models to modeling building (active learning) and predictive modeling [55,66,67,68]. Finally, although it is a challenging and complex optimization problem, it is worth investigating path planning for our sensor system in a variety of terrains.

## 7. Conclusions

In this work, we propose a soil monitoring framework characterized by heterogeneous sensor networks consisting of stationary and mobile sensors. The framework is centered on predictive modeling built using an active learning model building process. This is to reflect on two important constraints that likely impede the process of bringing recent advances in robotics and predictive modeling to soil monitoring. Soil monitoring is likely in the tool set of a diverse range of users because soil provides ecosystem services to mankind. These users have different application requirements. It is a challenge to meet the requirements. Another constraint is that because of the complex geological, chemistry, and physical process embedded in soil process, it is rare that we can fully understand the complex process and the users may also gradually learn what they need. The proposed framework thus provides great flexibility to meet diverse user requirements, and via an iterative active learning process, the framework gradually accommodate users’ evolving requirements, newly acquired sensing devices, and integrate user’s new understanding.

Within this framework, we implement a system that via active learning to select locations to deploy mobile sensors and to build three types of predictive models. The system demonstrates the feasibility and the promise of the framework.

Additionally, the numerical experiments with the system also lend us worthy lessons. First, naively adding more sensed data may not improve the performance of predictive models. Second, the combined model integrating interpolation and prediction leads to the best predictive performance.

## Figures and Tables

**Table 1:** Robot platform and sensor characteristics [15,41,42,43,44,45,46,47,48].

Robot Platform					
Robot Dimension	Speed	Climbing Slope	Lidar range	Endurance	Payload
50×43×25 cm	2.0 m/s	$0-20^{\circ }$	200 m	4 hour	20 kg
**Sensor Characteristics**					
Soil Sensor		Mechanism	Soil Properties		Remark
Salinity Sensor		Electrical	Salinity		Compact
pH Sensor		Electrical	pH Level		Compact
Density		Mechanical	Soil density		Compact
Nitrogen		Optical	Soil nitrogen		Compact
VH400 Moisture Sensor		Electrical	Water content		Portable, submerging
NDVI canopy sensor		Optical-Electrical	Radiometer		Compact
Agilent 4210 MP-AES		Plasma-spectral	Metal analyzer		Laboratory
GEN3 LITE		Sampling Robot arm	Samples collection		Portable

**Table 2:** Heavy metal concentration (ppm) statistics of Meuse data set.

Heavy Metal	Minimum	Maximum	Mean	Median	Standard Deviation
Cadmium	0.20	18.10	3.25	2.10	3.51
Copper	14.00	128.00	40.32	31.00	23.60
Lead	37.00	654.00	153.36	123.00	110.96
Zinc	113.00	1839.00	469.72	326.00	365.89

**Table 3:** Standalone spatial interpolation model via Gaussian process.

Heavy Metal	Training Data		Evaluation Data	
	$R^{2}\pm \sigma$	MAE ±σ	$R^{2}\pm \sigma$	MAE ±σ
Cadmium	0.89 ± 0.02	0.76 ± 0.08	0.44 ± 0.20	1.61 ± 0.36
Copper	0.89 ± 0.02	5.46 ± 0.47	0.52 ± 0.16	11.10 ± 2.05
Lead	0.88 ± 0.03	24.89 ± 2.99	0.47 ± 0.23	49.81 ± 10.00
Zinc	0.87 ± 0.01	90.19 ± 6.52	0.57 ± 0.17	152.98 ± 31.75

**Table 4:** Standalone prediction model via Gaussian process.

Heavy Metal	Training Data		Evaluation Data	
	$R^{2}\pm \sigma$	MAE ±σ	$R^{2}\pm \sigma$	MAE ±σ
Cadmium	0.84 ± 0.03	0.83 ± 0.08	0.77 ± 0.08	1.00 ± 0.20
Copper	0.85 ± 0.02	6.08 ± 0.41	0.77 ± 0.08	7.49 ± 1.27
Lead	0.87 ± 0.02	26.15 ± 2.15	0.59 ± 0.13	46.01 ± 6.85
Zinc	0.86 ± 0.01	90.74 ± 5.44	0.74 ± 0.07	122.63 ± 18.13

**Table 5:** Combined spatial interpolation and prediction model via Gaussian process.

Heavy Metal	Training Data		Evaluation Data	
	$R^{2}\pm \sigma$	MAE ±σ	$R^{2}\pm \sigma$	MAE ±σ
Cadmium	0.88 ± 0.01	0.73 ± 0.06	0.78 ± 0.08	0.98 ± 0.21
Copper	0.86 ± 0.02	5.79 ± 0.47	0.76 ± 0.09	7.55 ± 1.35
Lead	0.92 ± 0.01	20.33 ± 0.97	0.67 ± 0.11	39.80 ± 7.95
Zinc	0.90 ± 0.01	75.51 ± 4.62	0.75 ± 0.08	114.66 ± 23.37

## Data Availability

The MEUSE dataset is initially introduced by Burrough and McDonnell [49], and is publicly available via several packages of statistical programming language R, such as the sp and the gstat packages [51,52].

## References

[1] Amundson R., Berhe A.A., Hopmans J.W., Olson C., Sztein A.E., Sparks D.L. (2015). Soil and human security in the 21st century. Science.

[2] Pereira P., Bogunovic I., Muñoz-Rojas M., Brevik E.C. (2018). Soil ecosystem services, sustainability, valuation and management. Curr. Opin. Environ. Sci. Health.

[3] Kopittke P.M., Menzies N.W., Wang P., McKenna B.A., Lombi E. (2019). Soil and the intensification of agriculture for global food security. Environ. Int..

[4] Krause A., Singh A., Guestrin C. (2008). Near-optimal sensor placements in Gaussian processes: Theory, efficient algorithms and empirical studies. J. Mach. Learn. Res..

[5] Terzis A., Musaloiu-E R., Cogan J., Szlavecz K., Szalay A., Gray J., Ozer S., Liang C.J., Gupchup J., Burns R. (2010). Wireless sensor networks for soil science. Int. J. Sens. Netw..

[6] Wang J., Damevski K., Chen H. (2015). Sensor data modeling and validating for wireless soil sensor network. Comput. Electron. Agric..

[7] Lloret J., Sendra S., Garcia L., Jimenez J.M. (2021). A wireless sensor network deployment for soil moisture monitoring in precision agriculture. Sensors.

[8] Wang B. (2011). Coverage problems in sensor networks: A survey. ACM Comput. Surv. (CSUR).

[9] Elhabyan R., Shi W., St-Hilaire M. (2019). Coverage protocols for wireless sensor networks: Review and future directions. J. Commun. Netw..

[10] Xiao Y., Chen H., Wu K., Sun B., Zhang Y., Sun X., Liu C. (2009). Coverage and detection of a randomized scheduling algorithm in wireless sensor networks. IEEE Trans. Comput..

[11] Nguyen N.T., Liu B.H. (2018). The mobile sensor deployment problem and the target coverage problem in mobile wireless sensor networks are NP-hard. IEEE Syst. J..

[12] Luo W., Nam C., Kantor G., Sycara K. Distributed environmental modeling and adaptive sampling for multi-robot sensor coverage. Proceedings of the 18th International Conference on Autonomous Agents and MultiAgent Systems.

[13] Yang R.M., Guo W.W. (2019). Modelling of soil organic carbon and bulk density in invaded coastal wetlands using Sentinel-1 imagery. Int. J. Appl. Earth Obs. Geoinf..

[14] Bertalan L., Holb I., Pataki A., Szabó G., Szalóki A.K., Szabó S. (2022). UAV-based multispectral and thermal cameras to predict soil water content—A machine learning approach. Comput. Electron. Agric..

[15] Adamchuk V.I., Biswas A., Huang H.H., Holland J.E., Taylor J.A., Stenberg B., Wetterlind J., Singh K., Minasny B., Fidelis C., Kerry R., Escolà A. (2021). Soil Sensing. Sensing Approaches for Precision Agriculture.

[16] Ilčev S.D. (2019). Satellite Remote Sensing in Meteorology. Global Satellite Meteorological Observation (GSMO) Applications: Volume 2.

[17] Mani P.K., Mandal A., Biswas S., Sarkar B., Mitran T., Meena R.S., Mitran T., Meena R.S., Chakraborty A. (2021). Remote Sensing and Geographic Information System: A Tool for Precision Farming. Geospatial Technologies for Crops and Soils.

[18] Arrouays D., McBratney A., Bouma J., Libohova Z., Richer-de Forges A.C., Morgan C.L., Roudier P., Poggio L., Mulder V.L. (2020). Impressions of digital soil maps: The good, the not so good, and making them ever better. Geoderma Reg..

[19] McBratney A.B., Santos M.M., Minasny B. (2003). On digital soil mapping. Geoderma.

[20] Khaledian Y., Miller B.A. (2020). Selecting appropriate machine learning methods for digital soil mapping. Appl. Math. Model..

[21] Settles B. (2009). Active Learning Literature Survey. Technical Report 1648, University of Wisconsin–Madison Department of Computer Sciences. http://digital.library.wisc.edu/1793/60660.

[22] Ren P., Xiao Y., Chang X., Huang P.Y., Li Z., Gupta B.B., Chen X., Wang X. (2021). A survey of deep active learning. ACM Comput. Surv. (CSUR).

[23] Bektas T. (2006). The multiple traveling salesman problem: An overview of formulations and solution procedures. Omega.

[24] Kavraki L.E., Svestka P., Latombe J.C., Overmars M.H. (1996). Probabilistic roadmaps for path planning in high-dimensional configuration spaces. IEEE Trans. Robot. Autom..

[25] Woosley B., Dasgupta P., Rogers J.G., Twigg J. (2021). Multi-robot goal conflict resolution under communication constraints using spatial approximation and strategic caching. Robot. Auton. Syst..

[26] Bosse M., Newman P., Leonard J., Teller S. (2004). Simultaneous localization and map building in large-scale cyclic environments using the Atlas framework. Int. J. Robot. Res..

[27] Castellanos J.A., Martinez-Cantin R., Tardós J.D., Neira J. (2007). Robocentric map joining: Improving the consistency of EKF-SLAM. Robot. Auton. Syst..

[28] Cunningham A., Paluri M., Dellaert F. (2010). DDF-SAM: Fully distributed SLAM using constrained factor graphs. Proceedings of the 2010 IEEE/RSJ International Conference on Intelligent Robots and Systems.

[29] Bird B., Griffiths A., Martin H., Codres E., Jones J., Stancu A., Lennox B., Watson S., Poteau X. (2018). A robot to monitor nuclear facilities: Using autonomous radiation-monitoring assistance to reduce risk and cost. IEEE Robot. Autom. Mag..

[30] Sathyamoorthy A.J., Patel U., Guan T., Manocha D. (2020). Frozone: Freezing-free, pedestrian-friendly navigation in human crowds. IEEE Robot. Autom. Lett..

[31] Wadoux A.M.C., McBratney A.B. (2021). Digital soil science and beyond. Soil Sci. Soc. Am. J..

[32] Hassan-Esfahani L., Torres-Rua A., Jensen A., McKee M. (2015). Assessment of surface soil moisture using high-resolution multi-spectral imagery and artificial neural networks. Remote Sens..

[33] Peng Y., Xiong X., Adhikari K., Knadel M., Grunwald S., Greve M.H. (2015). Modeling soil organic carbon at regional scale by combining multi-spectral images with laboratory spectra. PLoS ONE.

[34] Sungmin O., Orth R. (2021). Global soil moisture data derived through machine learning trained with in situ measurements. Sci. Data.

[35] Murphy K.P. (2022). Probabilistic Machine Learning: An Introduction.

[36] Duvenaud D. (2014). Automatic Model Construction with Gaussian Processes. Ph.D. Thesis.

[37] Williams C.K., Rasmussen C.E. (2006). Gaussian Processes for Machine Learning.

[38] Neubeck A., Van Gool L. (2006). Efficient non-maximum suppression. Proceedings of the 18th International Conference on Pattern Recognition (ICPR’06).

[39] Survey U.G. National Geologic Map Database. https://en-us.topographic-map.com/.

[40] Rowell D.L. (2014). Soil Science: Methods & Applications.

[41] Owe M., de Jeu R., Walker J. (2001). A methodology for surface soil moisture and vegetation optical depth retrieval using the microwave polarization difference index. IEEE Trans. Geosci. Remote Sens..

[42] Adamchuk V.I., Hummel J.W., Morgan M.T., Upadhyaya S.K. (2004). On-the-go soil sensors for precision agriculture. Comput. Electron. Agric..

[43] Hemmat A., Adamchuk V. (2008). Sensor systems for measuring soil compaction: Review and analysis. Comput. Electron. Agric..

[44] Valente A. (2017). MEMS Devices in Agriculture. Advanced Mechatronics and MEMS Devices II.

[45] Kargas G., Soulis K.X. (2019). Performance evaluation of a recently developed soil water content, dielectric permittivity, and bulk electrical conductivity electromagnetic sensor. Agric. Water Manag..

[46] Naderi-Boldaji M., Tekeste M.Z., Nordstorm R.A., Barnard D.J., Birrell S.J. (2019). A mechanical-dielectric-high frequency acoustic sensor fusion for soil physical characterization. Comput. Electron. Agric..

[47] Chugh B., Thakur S., Singh A.K., Joany R., Rajendran S., Nguyen T.A., Denizli A., Nguyen T.A., Rajendran S., Yasin G., Nadda A.K. (2022). Electrochemical sensors for agricultural application. Nanosensors for Smart Agriculture.

[48] Vaz C.M.P., Porto L.F., D’Alkaine C.I., Bassoi L.H., Neto A.T., Hopmans J.W., Rolston D.E. (2022). Design and characterization of a pneumatic micro glass beads matrix sensor for soil water potential threshold control in irrigation management. Irrig. Sci..

[49] Burrough P.A., McDonnell R.A. (1998). Principles of Geographical Information Systems.

[50] Pebesma E.J. (2004). Multivariable geostatistics in S: The gstat package. Comput. Geosci..

[51] Pebesma E.J., Bivand R.S. (2005). Classes and methods for spatial data in R. R News.

[52] Gräler B., Pebesma E., Heuvelink G. (2016). Spatio-Temporal Interpolation using gstat. R J..

[53] Hengl T., Nikolić M., MacMillan R. (2013). Mapping efficiency and information content. Int. J. Appl. Earth Obs. Geoinf..

[54] D’Urso P., Vitale V. (2020). A robust hierarchical clustering for georeferenced data. Spat. Stat..

[55] Møller A.B., Beucher A.M., Pouladi N., Greve M.H. (2020). Oblique geographic coordinates as covariates for digital soil mapping. Soil.

[56] Hengl T. (2009). A Practical Guide to Geostatistical Mapping.

[57] Chicco D., Warrens M.J., Jurman G. (2021). The coefficient of determination R-squared is more informative than SMAPE, MAE, MAPE, MSE and RMSE in regression analysis evaluation. PeerJ Comput. Sci..

[58] Draper N.R., Smith H. (1998). Applied Regression Analysis.

[59] Glantz S.A., Slinker B.K., Neilands T.B. (2001). Primer of Applied Regression & Analysis of Variance, Ed.

[60] Pedregosa F., Varoquaux G., Gramfort A., Michel V., Thirion B., Grisel O., Blondel M., Prettenhofer P., Weiss R., Dubourg V. (2011). Scikit-learn: Machine Learning in Python. J. Mach. Learn. Res..

[61] Arlot S., Celisse A. (2010). A survey of cross-validation procedures for model selection. Stat. Surv..

[62] Liu H., Ong Y.S., Shen X., Cai J. (2020). When Gaussian process meets big data: A review of scalable GPs. IEEE Trans. Neural Networks Learn. Syst..

[63] Pouyanfar S., Sadiq S., Yan Y., Tian H., Tao Y., Reyes M.P., Shyu M.L., Chen S.C., Iyengar S.S. (2018). A survey on deep learning: Algorithms, techniques, and applications. ACM Comput. Surv. (CSUR).

[64] Hengl T., Nussbaum M., Wright M.N., Heuvelink G.B., Gräler B. (2018). Random forest as a generic framework for predictive modeling of spatial and spatio-temporal variables. PeerJ.

[65] Sekulić A., Kilibarda M., Heuvelink G.B., Nikolić M., Bajat B. (2020). Random forest spatial interpolation. Remote Sens..

[66] Heuvelink G.B., Pebesma E.J. (1999). Spatial aggregation and soil process modelling. Geoderma.

[67] Vereecken H., Schnepf A., Hopmans J.W., Javaux M., Or D., Roose T., Vanderborght J., Young M., Amelung W., Aitkenhead M. (2016). Modeling soil processes: Review, key challenges, and new perspectives. Vadose Zone J..

[68] Behrens T., Schmidt K., Viscarra Rossel R.A., Gries P., Scholten T., MacMillan R.A. (2018). Spatial modelling with Euclidean distance fields and machine learning. Eur. J. Soil Sci..

